# General Practitioners’ Perceptions of Heat Health Impacts on the Elderly in the Face of Climate Change—A Qualitative Study in Baden-Württemberg, Germany

**DOI:** 10.3390/ijerph15050843

**Published:** 2018-04-24

**Authors:** Alina Herrmann, Rainer Sauerborn

**Affiliations:** 1 Institute of Public Health Heidelberg, University Hospital Heidelberg, 69120 Heidelberg, Germany; rainer.sauerborn@urz.uni-heidelberg.de; 2 Network Aging Research, University of Heidelberg, 69115 Heidelberg, Germany

**Keywords:** general practitioners, elderly, heat, heatwave, climate change, perceptions, adaptation

## Abstract

Heat health impacts (HHI) on the elderly are a growing concern in the face of climate change and aging populations. General practitioners (GPs) have an important role in health care for the elderly. To inform the development of effective prevention measures, it is important to investigate GPs’ perceptions of HHI. Twenty four qualitative expert interviews were conducted with GPs and analyzed using the framework approach. GPs were generally aware of heat health impacts, focusing on cardiovascular morbidity and volume imbalances. Perceptions of mortality and for instance impacts on respiratory diseases or potentially risky drugs in heat waves partly diverged from findings in literature. GPs judged the current relevance of HHI differently depending on their attitudes towards: (i) sensitivity of the elderly, (ii) status of nursing care and (iii) heat exposure in Baden-Württemberg. Future relevance of HHI was perceived to be increasing by most GPs. The main cause identified for this was population aging, while impacts of climate change were judged as uncertain by many. GPs’ perceptions, partly diverging from literature, show that GPs’ knowledge and awareness on HHI and climate change needs to be strengthened. However, they also emphasize the need for more research on HHI in the ambulant health care setting. Furthermore, GPs perceptions suggest that strong nursing care and social networks for elderly are major elements of a climate resilient health system.

## 1. Introduction

In the heat wave summer of 2003, Baden-Württemberg experienced about 900–1300 and Europe for up to 70,000 excess deaths due to extreme temperatures [1,2]. Deaths attributable to heat waves are projected to rise significantly in Germany as in other European countries until the end of the 21st century due to climate change and aging populations [3,4]. There is no universally accepted definition of a heat wave [5]. However, in public health research heat wave definitions focus on their impacts on human health and often describe periods of at least 48 h, in which temperatures exceed a certain threshold (for instance 32° Celsius in Germany) [6,7]. These definitions often consider apparent or perceived temperature, which also factors in variables such as humidity, which influence temperature regulation [8,9]. Zacharias et al. projected a tripling of the frequency of heat waves and a 25% increase in the duration of heat waves in Germany by the end of the century compared to the present climate leading to a rise in excess deaths from ischemic heart disease due to heat waves by a factor of 2.4 [10]. The elderly are particularly vulnerable to health impacts from heat waves [11,12]. The elderly are defined as persons aged over 75 years in this study, because this age group has been found to be particularly vulnerable to heat in Baden-Württemberg [13]. On the one hand, age subsumes many risk factors for suffering from heat such as pre-existing somatic and mental diseases, dependency on nursing care and intake of medication [11,14,15,16]. A broad range of drugs have been identified to pose a threat in heatwaves because of interfering with the ability to adapt to heat (see Table 1). On the other hand, some physiological changes of aging itself can also pose a risk in the absence of disease [17] such as a reduced number of cutaneous blood vessels or higher temperature thresholds for onset of sweating [18,19].

In this study, heat health impacts are defined as incident cases of diseases and deaths which can be attributed to heat waves, as defined by the heat alert system of the German Weather Service (38 °C on one day or 32 °C on two following days without sufficient cool-down at night) [7]. Heat health impacts don’t only comprise specific heat-related conditions such as heat stroke. Common other causes of excess death in heat waves in Germany are cardiovascular and respiratory diseases [20,21]. Furthermore, studies in other countries, looking at the general relationship between heat and mortality found cerebrovascular mortality to increase with heat [22]. Moreover, health care utilization increases during heat waves: two large-scale multicenter studies in the US and Europe, examining the increased morbidity on hot days and heatwaves, reported that hospitalization and emergency visits increased for respiratory and renal, but not for cardiovascular disease [22,23].

According to Astrom et al. even just to maintain heat-related mortality at present levels, substantial adaptation efforts are needed [4]. Heat health action plans need to be developed and implemented in Germany and elsewhere to prevent adverse heat health impacts [24]. However, heat health action plans are not known by both practitioners and the population, particularly the elderly [25]. In order to engage stakeholders in preventive action and develop feasible health interventions, including heat health action plans, it is important to examine the perceptions of stakeholders on the underlying health issue [26,27]. According to the WHO, heat health action plans should be designed with stakeholders at all levels [28], including general practitioners (GPs). This is because GPs are valued as trusted persons, who take a central role in giving heat health advice and promoting preventive behavior [29,30]. Furthermore, they can take on a unique role in reaching the elderly, especially those who are socially isolated. For many socially isolated people, their GP is one of the few social contacts they have [31]. In addition, adjusting medication during heat waves is a task which is exclusively tied to the profession of medical doctors [11,28].

Although there is a broad range of responsibilities, which GPs can possibly take on in heat health action plans, their perceptions of heat health impacts have been investigated very rarely so far. So far only a few GPs have been enrolled in studies which have investigated perceptions on heat health risks and heat health action plans among health professionals [25,32,33,34]. From the little we know, GPs in Australia were aware that heat can pose a health threat. Their knowledge on thermoregulation and heat–related illness was reported to be good [33]. In a survey on extreme weather events with community based physicians in Germany, 77 percent of the participants thought that traffic hindrance through snow and ice on roads had the strongest impact on their work, whereas only 25 percent thought so regarding heat, although multiple answers were possible. Fifty-one percent agreed that flu epidemics would be a greater challenge than extreme weather events [35]. Thus, in the few studies we have, physicians don’t seem to attribute a great health relevance to extreme weather events in general and heat waves in particular.

To our knowledge this is the first study to investigate general practitioners’ perceptions of heat health impacts on the elderly and on the impacts potential links with climate change. In particular, we studied GPs’ perceptions of the definition of a heat wave, their perceptions of risk factors for suffering from heat as well as how heat impacts morbidity and mortality of their elderly patients. Furthermore, we studied what clinical relevance GPs attribute to heatwaves now and in the future and why.

## 2. Materials and Methods

**Figure 1:**
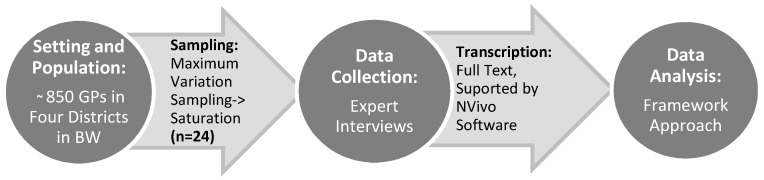
Overview of the study design. The Setting BW stands for Baden-Württemberg. Expert interview were conducted according to the method of Meuser and Nagel [46] and the framework approach for the data analysis followed the methodology of Ritchie and Spencer [47].

This study is part of a bigger research project, which investigates possible measures for general practitioners to prevent heat health impacts for the elderly in Baden-Württemberg, Germany. In order to develop feasible prevention measures the research project makes use of implementation frameworks, which describe relevant factors for implementation on organizational, structural, intervention, patient and provider level [27,36,37]. In the results presented in this paper we focus on the provider level. For providers of health interventions, in our case GPs, factors such as knowledge and attitudes or perceived need for an intervention are factors relevant for implementation [27,36,37]. We chose to apply qualitative methodology, because qualitative methods are particularly useful to thoroughly understand perceptions and attitudes of individuals [38]. In particular, we conducted qualitative expert interviews with GPs. Figure 1 summarizes the steps of recruitment of participants, data collection and data analysis.

The study setting comprised four administrative districts in the Rhine-Neckar Region of Baden-Württemberg, Germany (namely *Stadt Heidelberg, Stadt Mannheim, Neckar-Odenwald-Kreis* and *Rhein-Neckar-Kreis*). Those districts were chosen because they lay in or close to the upper Rhine Valley, one of the regions with greatest heat stress in Germany [39]. Furthermore, they differ in population density, income, and share of migrants [40,41,42]. Thus, a wide variety of social realities was covered. As source population, we considered all 850 GPs of these districts registered with the Association of Statutory Health Insurance Physicians of the state of Baden-Württemberg early 2013 (*Kassenärztliche Vereinigung BW*) [43]. With a purposeful, maximum variation sampling technique, we sampled GPs with different characteristics in order to cover a great variation of perspectives [44]. Those characteristics comprised GP’s sex, age and specialization (general medicine, internal medicine) as well as different practice settings (urban and rural—rural also comprised suburban settings) and practice structures (single practice, joint practice, ambulatory health care center).

We recruited general practitioners from May to August 2013 and conducted 22 interviews during that time period and another two interviews in September and October 2013 until data saturation was reached [45]. Overall 51 practices were contacted, which makes a response rate of 47%. We purposely chose to collect the data for several months during the pre-heat and heat season. First, the seasonal timing made it easier for GPs to relate to the topic. Second, the long duration allowed us to transcribe the interviews and familiarize with the data in parallel to data collection, which enabled us to detect the right point of saturation. A high grade of repetition of important topics was reached after 22 interviews and we could not detect new themes. However, we conducted another two interviews in September and October 2013 to make sure, that no new themes were coming up.

Data collection was accomplished by using qualitative expert interviews [47,48]. The expert interviews were conducted as semi-structured interviews in GPs’ offices. In terms of content, the development of the interview guide for this study was guided by the research objectives (see above). Furthermore, inputs from the literature review were taken into account. The guiding principles in phrasing the interview questions were *openness, neutrality*, *clarity and simplicity* as proposed by Gläser and Laudel [48]. The interview guide can be found in the Appendix A (Table A1). The interview guide also contains questions dealing with *prevention* of heat health impacts for the elderly. Results on prevention will be published in a separate article and are not contained in the analysis described here.

Ethical clearance for this study was attained from the ethical board II of the medical faculty of Heidelberg University (Sign S-130/2013). All GPs were informed about the study objectives and the research procedures including the recording of interviews. They documented their approval by signing the informed consent. GPs were informed that study participation was entirely voluntary and that they could withdraw at any time. The interviews lasted half on hour on average and were recorded and transcribed verbatim according to a transcription scheme developed by Dresing and Peel [49].

The analysis was accomplished using the framework approach [46]. The framework approach is a qualitative content analysis technique developed for applied policy research, which focuses on output that can be used to develop or improve policy measures [46]. We applied the five steps of the framework approach in the following way: The familiarization (1) was accomplished during data transcription as it was only one researcher (AH) to conduct and transcribe all interviews. Afterwards we analysed the data by developing a thematic framework (2) and indexing (= coding) the transcripts (3). At this stage we defined four main themes. Theme A, B, and C are described separately in the results section, while the so-called theme 0 contains the first thoughts of GPs on the topic and describes important overarching aspects. The four themes and an overview of first and second level codes are displayed in Supplementary Materials. As all interviews were conducted, transcribed and analyzed in German, we decided to keep these original documents in German. The first three steps of analysis were supported by the software NVivo [50]. The results of the more abstract steps of charting (4) and mapping and interpretation (5) were done without NVivo and are displayed in the results section.

## 3. Results

The results section starts with GPs’ perceptions of heat wave characteristics (Theme A). Afterwards, it will explore GPs’ perceptions of heat-related morbidity and mortality in the elderly, as experienced in their daily routine (Theme B). Finally, it will describe how GPs judged the current and future relevance of heat health impacts (Theme C).

### 3.1. GPs’ Perceptions of Heat Wave Characteristics

**Figure A1:**
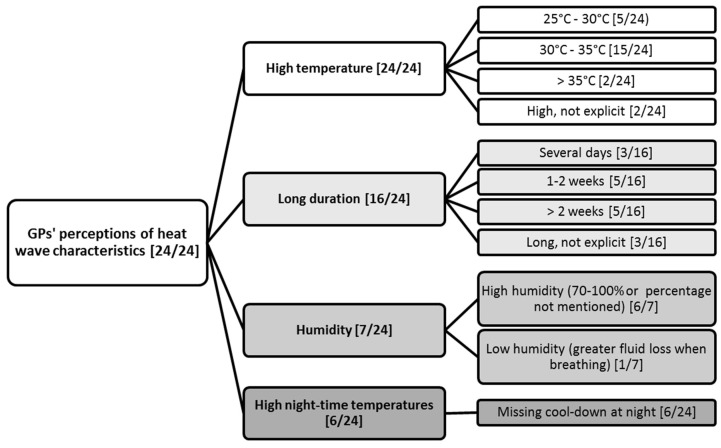
GPs’ perceptions of heat wave characteristics.

To start with, we openly asked GPs what they thought a heat wave to look like without prompting for certain characteristics. GPs commonly used four categories to describe their perceptions of heat wave characteristics: high temperature [24/24], long duration [16/24], humidity [7/24] and high night-time temperatures [6/24]. Most GPs thought temperatures of 30–35 °C to be characteristic for a heat wave [15/24]. Those GPs considering temperatures below 30 °C to be sufficient for a heat wave definition [6/24], always tied an additional criterion to it, such as duration of more than a week, high humidity or high night-time temperatures. Some GPs considered humidity as criterion for a heat wave [7/24]. Most GPs, who mentioned duration as a characteristic, thought heat waves to last at least for one or two weeks [10/16] (see also Figure A1 in the Appendix A).

As mentioned in the introduction the German Weather Service gives out heat warnings when the perceived temperature exceeds 32 °C on two consecutive days with a marginal cooling down at night. The perceived temperature considers air temperature, humidity and other variables such as wind speed and mean radiant temperature [8]. After being prompted with the German Weather Service definition some of the GPs were surprised about this short time period. One GP said, that two days might be justified, when there was extreme heat stress (GP 12). Furthermore, one GP insisted, that not the absolute temperature, but the elderlies’ social support and nursing care situation were crucial (GP 6).

### 3.2. GPs’ Perceptions of Risk Factors to Suffer from Heat Health Impacts

As risk factors to suffer from heat health impacts GPs mentioned pre-existing diseases [23/24], socioeconomic factors [19/24], individual factors [18/24], intake of medication [14/24], functional impairments [5/24] and alcohol intake [2/24] (see also Table A2 in the Appendix A).

#### 3.2.1. Pre-existing Disease 

For pre-existing diseases, almost all interviewees mentioned cardiovascular diseases as risk factors, because heat could aggravate cardiovascular complaints [15/23]. Several also identified dementia and other cognitive impairments [8/24] to make the elderly less capable to detect a risky situation and react adequately. Examples for the lack of adequate behavioral adaptation were identified in insufficient fluid intake and lack of systematic planning (e.g., lack of proper ventilation). Only one quarter of GPs mentioned respiratory diseases [6/24]. Furthermore, GPs emphasized that multi-morbidity increased the risk of heat health impacts [8/24].

#### 3.2.2. Socioeconomic Factors

The majority of GPs thought socioeconomic risk factors, such as the social support and nursing care situation to be crucial when judging the risk of the elderly to suffer from heat [19/24]. For instance, living alone without support was judged to be a risk factor [11/24]. Many GPs argued that quality of formal or informal care as well as the availability of a social network were decisive for the risk to experience heat health impacts. This was because such stakeholders could support elderly in adapting their behavior, e.g., by reminding them to drink sufficiently or by supporting them in activities of daily living in order to reduce physical activity and heat exposure:

“If they are living alone, I think they are more at risk, because no one is looking after them. If the family lives with them […], one will look after them. In care homes, for example, they take care that there is sufficient drink and that they don’t go outside, especially in the greatest heat.”

*GP 11 (female, rural, 51, general medicine, single practice)*

#### 3.2.3. Individual Factors

The most important individual factor mentioned was age. Many GPs felt, that one of the biggest problems with age was the reduced fluid intake of elderly. Yet, it was also mentioned that age was an insufficient criterion to judge the risk of suffering from heat:

“There are 75 year olds that are fit as a fiddle. So you really can’t lump them together […], others are already frail with 65.”

*GP 6 (female, urban, 50, general medicine, joint practice)*

#### 3.2.4. Intake of Medication

Most GPs mentioned medication intake to pose a health threat in heat episodes [14/24]. Those 14 GPs were not prompted with drugs as a risk factor, but came up with drugs as risk factor themselves. Antihypertensive drugs such as beta-blockers and calcium antagonists, as well as diuretics, were mentioned most often [13/24]. These drugs were said to lead to circulatory problems like fainting, increase the risk of dehydration and develop electrolyte imbalances. GP 10, for instance, reasoned that diuretics caused vasodilation and increased water excretion, thus bearing the risk of hypotension.

### 3.3. GPs’ Perceptions of Morbidity and Mortality as Heat Health Impacts

When asked about their perceptions of heat health impacts on their elderly patients, some GPs conceded that they had not been confronted much with heat health impacts in their offices. Several GPs argued that heat health impacts were often not explicit. The elderly might suffer from heat, but heat health impacts would not necessarily be the reason to visit the doctor:

“Well they [the patients] usually come anyways due to blood pressure or due to diabetes or due to circulatory problems, which they always have. And the weather, […] that can intensify the complaints, but that won’t be the cause for the people to come.”

*GP 11 (female, rural, 51, general medicine, single practice)*

Furthermore, few GPs proposed that little experience of their patients’ heat health impacts might be due to the pattern of health care utilization. For instance, GPs thought that people would rather stay at home during hot weather or if they suffered severe problems might be brought into the emergency units right away. One GP uttered that heat health impacts did not exist in Germany (GP 4). He argued that health care and provision with food and drinks in Germany were sufficient to counteract any heat health impacts. However, all other GPs did at least report about some heat health impacts.

#### 3.3.1. Morbidity as Heat Health Impact

All GPs described morbidity related to heat. Perceptions of heat-related morbidity were mostly centered around volume depletion and electrolyte imbalances [22/24] as well as circulatory diseases [15/24] and effects of heat and light [8/24]. In comparison, other disorders, such as infectious diseases [7/24] or mental disorders [5/24] were mentioned less often with renal and respiratory diseases only mentioned sporadically. A more detailed description of the diagnoses mentioned by GPs is given in the Appendix A, coded according to ICD-10 (Table A3 in the Appendix A).

#### 3.3.2. Mortality as Heat Health Impact

**Figure 2:**
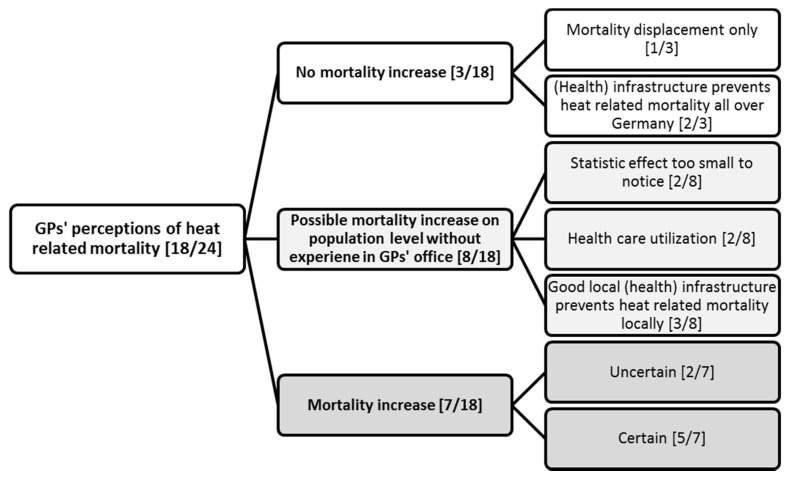
GPs’ perception of heat related mortality.

When asked about heat health impacts, GPs seldom associated heat waves with mortality spontaneously. As we noticed this throughout the data collection phase, we specified our question, asking for impacts on morbidity and mortality explicitly in the subsequent interviews. Thus, death as a heat health impact was only discussed with 18 of 24 GPs. Within this group of 18 GPs, perception of mortality in heat waves varied and it was often distinguished between GPs’ experience in their daily work and epidemiological findings (see Figure 2).

Seven out of eighteen GPs associated heat waves with an increase in mortality. Most of these GPs were certain about this, whereas some expressed uncertainty. However, even those GPs did not attribute one of their own patients’ death clearly to a heat wave. Furthermore, none of the GPs drew on epidemiological evidence to support their argument:

“Well, when there are really such heat waves, like e.g., in 2003, then I can imagine, I am even convinced that then a lot of elderly people, who don’t really have the opportunity to adapt through foods or fluids or something like that, that then mortality rises, especially in nursing homes. […] I don’t know if there’s literature about this.”

*GP 12 (male, rural, 49, general medicine, single practice)*

Eight out of eighteen GPs thought that a mortality increase was possible at the population level, but stated, that they themselves had never experienced such an increase. Some thought that this was due to statistical reasons, because the number of total deaths was too small to be notice in a single practice.

“I don’t know how many GPs there are in Baden-Württemberg? (…) There should be more than thousand, shouldn’t there? If we distribute 1100 more deaths on all the GPs then it would be about half a death more in the summer. You don’t notice that. You can only extricate it out of a statistic.”

*GP 7 (male, urban, 44, internal medicine, joint practice)*

Others felt that local circumstances made it less likely that an increase in mortality in heat would occur in their specific area of practice. For instance, one GP stressed that there would not be a mortality increase in her district, as it was a wealthy district with well-educated elderly that were well taken care of by formal or informal carers, including full-time care by Eastern European health professionals. Others reported, that they might only notice an increase in morbidity, not mortality because people would be brought to the clinic immediately, if they suffered from heat in a life-threatening way (see above).

Finally, three out of eighteen GPs explicitly rejected that heat waves increased mortality at all. They either said that they perceived the health care for elderly by informal or formal carers in Germany as sufficient to prevent excess heat mortality or that mortality displacement, also called the harvesting effect, would account for all excess death in a heatwave.

“It is definitely so, that—when someone is already very ill and there is a heat wave and you expect that the person will die soon anyways—then it happens a bit more quickly due to the heat […]. But not so, that there is a greater number of deaths through heat waves, I don’t think so.”

*(GP 22, female, rural, 44, internal medicine, joint practice)*

### 3.4. GPs’ Perceptions of Relevance of Heat Health Impacts for Elderly

#### 3.4.1. Current Relevance

We asked GPs how relevant they perceived heat health impacts regarding their patients’ overall situation. Some GPs attributed a low relevance to heat [6/24]. An equal number of GPs either attributed high relevance to heat [9/24] or had a balanced attitude on that topic [9/24]. All GPs made use of the same two rationales of sensitivity and exposure to explain why they perceived heat health impacts to be relevant or not. Exposure describes the intensity, duration or frequency of thermal stress facing a population while sensitivity is a function of the individual, demographic and socioeconomic characteristics that influence how individuals can deal with thermal stress [13]. Thus, the scheme of vulnerability as an interplay of sensitivity and exposure was addressed by GPs. Adaptation as the third aspect of vulnerability was addressed indirectly on the individual level, for instance when describing the ability of the elderly to adapt their behavior. However, as these aspects were very closely interlinked with the sensitivity and not put into the context of a structured adaptation approach by GPs, we subsumed aspects of adaptation under the category of sensitivity, similarly to Jendritzky, who also considers adaptation of the individual to be part of sensitivity [13].

Table 2 illustrates how the same rationales of sensitivity and exposure lead to different attribution of relevance. For instance, some GPs declared that nursing care needs were sufficiently met within the existing system while others judged current nursing care to be insufficient to meet special nursing care needs during heat waves:

“A great [relevance], because we already see it in practice, that the number of single elderly rises and that the neighborly support decreases, which used to be much better. I can see and judge this by my long experience. Yes and the same is the case in nursing homes that reduce staff as much as possible, as well as quality, in order to make it cheap. And thus nursing care there is not very good either.”

*GP 20 (male, urban, 62, GM, JP)*

#### 3.4.2. Future Relevance

**Figure 3:**
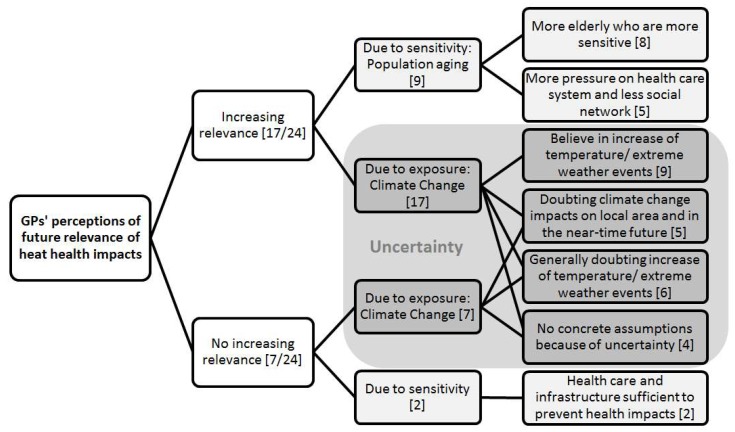
GPs’ perception of future relevance of heat health impacts on the elderly. Counts on the first level are exclusive [x/24], while counts on other levels [x] are not.

After talking about current relevance of heat for GPs’ elderly patients, GPs were asked whether they thought that this relevance would change in the future. Figure 3 summarizes in a graphic way why GPs perceive the future relevance of heat health impacts to increase or not.

Most GPs [17/24] judged the relevance to increase and some [7/24] judged that the relevance would not change. As in the question on current relevance the GPs talked about the topics of sensitivity and exposure. From the sensitivity perspective, population aging emerged to be the main topic of vulnerability when thinking about the future. From the exposure perspective, climate change was the topic which was brought up to be relevant by GPs.

#### 3.4.3. Increasing Sensitivity through Population Ageing

More than half of the GPs, who judged heat health impacts to be of increasing relevance, attributed this mainly or partly to the increasing sensitivity of the population caused by population aging [9/17]. Besides the sheer greater number of elderly, GPs pointed out that the social support and nursing care situation would deteriorate, thus rendering the elderly in the health system more sensitive. According to GPs this was the case either because of change in family structures (fewer children, family members not living in the same place) or because of increasing pressure on the system of professional nursing care:

Interviewer: “What role will this topic play in the future?”, GP: “Rather an increasing role. Not because of the heat alone, but simply because provision for the elderly comes more and more into focus, especially in the care sector. People just get older and older. This is a big question of staffing.”

*GP 12 (male, rural, 49, general medicine, single practice)*

Within the group of GPs that judged the relevance of heat health impacts not to be increasing [7/24], only two mentioned the sensitivity perspective. These two judged the health system to be sufficiently prepared to meet the additional health care needs during heatwaves.

None of the GPs commenting on the sensitivity perspective expressed uncertainty. For some of the GPs attributing an increasing relevance to heat health impacts in the future their main rationale to do so was the sensitivity perspective (population aging), not the exposure perspective (climate change).

“It is of course not exactly predictable, whether the summers will really become warmer […]. From that point of view, I only see the greater relevance in the fact, that generally people become older and that the part of people over 75-years rises. I wouldn’t, thinking of the last summer, which wasn’t very good, automatically think that all the summers in the future will be hotter. But the proportion of elderly people definitely rises.”

*GP 15 (female, urban, 51, internal medicine, single practice)*

#### 3.4.4. Increasing Exposure through Climate Change

Most GPs [21/24] mentioned the topic of climate change, when asked about the future relevance of heat health impacts, the rest was prompted with that topic [3/24]. However, their attitudes regarding climate change were very heterogeneous (see also Figure 3). Within the group of GPs who judged heat health impacts to be of increasing relevance, different perceptions prevailed. Most GPs in that group [9/17] stated that temperature and/or extreme weather events would increase.

“Well I think that we will have more heat waves due to climate change. And that you have to consider […] how to react to that. Well, I do think that the relevance will rise in the next few decades, because I think that extreme weather will just increase due to climate change. (…) It is possible that then a challenge will arise to care for all those people.”

*GP 23 (male, suburban, 39, internal medicine, medical care center)*

Still, when talking about climate change GPs often linked their statements with expressions of uncertainty [18/24]. Few GPs didn’t want to make concrete statements about meteorological changes caused by climate change at all because of uncertainty. GPs seemed to show less uncertainty, when talking about extreme weather events than when talking about rise in temperature. Furthermore, it was striking, that GPs usually referred to their immediate or long-term experience when evaluating climate change. If scientific information was mentioned, this was often done in a framing of mistrust.

“The other thing is that it is supposed to become warmer and warmer (laughs). But this happens slowly. Presently, I don’t have the impression. Now it’s raining all the time. So, we don’t really know. Maybe it’s getting warmer, maybe we get a rather tropical climate, so we get more rainfall. I am not an expert in these questions. I don’t really believe in these weather prognoses.”

*GP 3 (male, urban, 53, general medicine, joint practice)*

In the group of GPs that did not judge relevance of heat health impacts to increase, most GPs doubted increase of temperature and/ or extreme weather events in general. The other GPs in that group thought that climate change would either not have impacts locally (in Germany or in their region) or not have impacts in the near future. Two GPs described possible meteorological changes caused by climate change, but did not think that it could have any impacts on health.

### 3.5. Overarching Theme: Importance of Social Support and Nursing Care

In all sections of the results (Section 3.1, Section 3.2, Section 3.3 and Section 3.4) GPs emphasized the sensitivity perspective and underlined the importance of social support and nursing care in the ambulatory and stationary setting. From their perspective as health care providers the sensitivity perspective is more important than the exposure perspective. Furthermore, GPs identify social support and nursing care to be important elements to prevent heat health impacts for the elderly.

## 4. Discussion

### 4.1. Methodological Strengths and Limitations

To our knowledge this is the first qualitative study about perceptions of heat health impacts which concentrates on GPs only. In similar studies with different health care providers only very few GPs could be recruited. For instance, Abrahamson et al. sent letters to 177 GPs in three London boroughs and only one GP was ready to participate [51]. Ibrahim et al. sent invitations to about 6000 district GPs for a quantitative study and because they did not get any positive feedback, they included a convenience sample of 11 GPs [33]. Williams et al. included one GP on the basis of a personal contact [34].

In contrast to this, the GPs in the current study were recruited in a structured purposive maximum variation sampling, which was guided by a clearly pre-defined sampling framework. Furthermore, within the groups of framework characteristics, GPs were randomly chosen from a pre-defined source population (all GPs in the four included administrative districts) and the response rate was 47%.

When displaying our results, we decided to give counts about the number of mentions of certain codes, categories and themes. These counts should give an impression about the importance that was given to certain topics throughout the sample. As this is a qualitative study they do not allow drawing conclusions about the statistical distribution of certain attitudes among the source population of GPs. However, as we will see throughout the discussion the results fit findings from other research, indicating that general findings from this study are valid and transferable to similar circumstances [52].

### 4.2. GPs’ Perceptions of Heatwave Characteristics

Corroborating our results, Ibrahim et al. found that on average GPs and other health staff thought that the mean maximum daytime temperature representing a hot day was 31 °C [33]. Yet many of GPs in this study thought heat waves to last over a week compared to common definitions, which usually do not exceed five days of duration [53,54]. In the public health setting heat wave definitions start off with two days because research shows that lag times of heat health impacts are short, usually between 0–3 days [22]. Furthermore, only a forth of GPs mentioned high humidity or high night time temperature spontaneously, although this is part of the heat wave definition of most heat warning systems, including the German one [7,53,55]. Thus, in communication with GPs it is important to stress that heat waves can be deadly, even if they are short and even more so if humidity and night time temperatures are higher.

### 4.3. GP’s Perceptions of Risk Factors to Suffer from Heat Health Impacts

Almost all risk factors, which have been identified in the literature, have been covered in the presented interviews, when considering the answers of all GPs (see Table A2 in the Appendix A). Yet, within the category of pre-existing diseases most GPs focused on cardiovascular disease, while areas such as respiratory disease and diabetes were often neglected. Many studies report that cardiovascular diseases (CVD) in general or specific CVDs, such as coronary heart disease, conduction disorders or congestive heart failure, increase the risk of heat-related mortality [14,56,57]. Yet there is also some conflicting evidence. For instance, Schifano et al. found that previous hospitalization for cardiovascular disease had a protective effect in heat waves [16]. Neuropsychological or mental diseases were consistently found to be a risk factor for heat-related mortality and are even found to pose a greater risk than cardiovascular disease [14,16]. Still, most GPs named cardiovascular risk factors while only a quarter of them mentioned neuropsychological diseases as a risk factor. Also other diseases were hardly mentioned by GPs but have been found to increase heat related mortality. For instance, Zanobetti et al. showed that higher temperature in summer time led to shorter survival time in elderly patients with COPD (chronic obstructive pulmonary disease) and diabetes in a time series study with four cohorts from 135 US cities [57].

We observed the same tendency of GPs focusing on CVD in the perceptions of drugs that increase health risk during heatwave events. While the majority of GPs were aware that drugs can be a potential risk factor, GPs focused on drugs used in CVD treatment, such as diuretics, beta-blockers and calcium antagonists. If one compares the drugs defined as potentially risky in a review by Westaway et al. (Table 1) with the drugs mentioned by GPs (Table A2 in the Appendix A) one can see that most GPs were not aware of the full range of potentially risky drugs.

Many GPs stressed that a generally deteriorated health status, namely multimorbidity, functional impairments and a dependency on nursing care, poses a risk to suffer from heat. This is reflected in literature, as Belmin et al. could establish care level as a determining factor for susceptibility to heat and Vandentorren found reduced mobility was one of the greatest risk factors for deaths in the 2003 heat wave in France [14,58]. Moreover, GPs stressed the importance of social support and nursing care and valued good formal or informal nursing care as protective factor for the most susceptible populations. Some GPs even valued quality of care so highly, that they judged for instance living alone not to be a risk factor, if compensated by a caregiver. However, Vandentorren et al. found that having home attends did not mean a lower, but a higher risk of dying in a heatwave [14].

### 4.4. GPs’ Perceptions of Morbidity as Heat Health Impact

GPs’ mentions of heat-related diagnoses of morbidity did not fully match with the existing literature on heat morbidity, except for heat illnesses, such as volume depletion or electrolyte imbalances. For instance, renal failure, respiratory diseases or diabetes were found to be significantly increasing with temperature, but were hardly mentioned by our GPs [12,22]. One explanation for this discrepancy could be that existing literature on heat morbidities usually draws on hospitalization or emergency department visits, and hardly ever includes data of the ambulatory health care setting such as GP visits.

Smith et al. conducted two studies particularly focusing on the ambulatory health care setting by comparing GP in-hour visits, GP out-of-hour visits and emergency department visits in a heatwave year to non-heatwave years in the UK. They found that GP in-hour visits for heat illness increased during heatwaves in all age groups. For the 75+ population heat illness even tripled compared to non-heatwave years and rates of heat illness also stayed higher for some time after the final heat alert [59]. However, they did not find an increase for any other morbidity [60]. Smith and Elliot partly attributed this to the mildness of the investigated heatwave, but also discussed that elderly followed the advice to stay out of the heat and therefore did not go to see their doctor. These findings of Smiths and Elliot were mainly corroborated in our data, as described in chapter 3.3. Thus, GPs might either not notice or simply not be confronted with many of the heat health impacts, which are described in large epidemiological studies. In order to better understand discrepancy between GPs perceptions of morbidity and common findings in literature, which usually draws on hospitalization or emergency department visits, more research is needed on heat morbidity in the ambulant health sector.

### 4.5. GPs’ Perceptions of Mortality as Heat Health Impact

As depicted in Figure 2, many GPs [8/18] thought that mortality increase at the population level might be possible, but stated that they were not sure, because they did not experience this in their daily routine. As discussed in the introduction, many studies have shown that heat and heat waves increase mortality especially for the elderly [2,12,61]. On the one hand this leads to the conclusion that GPs need to be better informed about heat-related mortality. Obviously, they were not acquainted with the current literature in heat health impacts. However, the perceptions of general practitioners also invite researchers to look at the topic of heat health from a practitioner’s perspective. In the most severe heat wave in recent years in 2003, about 900 to 1300 excess deaths occurred in Baden-Württemberg [1]. Currently, 7000 GPs practice in Baden-Württemberg [62]. Thus, less than every fifth GP had experienced one excess death due to heat in the 2003 heat wave. This example illustrates why some GPs did not associate heat and mortality and rather focused on the underlying morbidity, when thinking about heat health impacts.

Other statements of GPs underline the importance of drawing a precise and differentiated picture of heat health impacts. For instance, some GPs were convinced that heat would not increase mortality, if the harvesting effect was taken into account. The “harvesting effect” describes a forward displacement of mortality from an expected later point of time to premature time of deaths during a heat event [63]. This harvesting effect can be observed statistically by looking for a reduction of deaths *after* heat events. Although the harvesting effect is known in research and studies acknowledge it, many studies don’t take it into account in their analysis [64,65]. For instance, Bacchini et al. did investigate the harvesting effect in the European PHEWE study, but decided to not consider the effect in their final results. Still, they call for a more rigorous consideration of the harvesting effect in future studies and propose to find more sophisticated ways of quantifying heat health impacts, for instance in a years of life lost analysis [66].

Some GPs pointed out that they could imagine that heat increases mortality, but that they believe that it does not in their district. Hondula et al. investigated spatial differences in mortality in Philadelphia County (PA, USA). They found that while measuring a countywide excess mortality of 5.5% above a threshold of a certain apparent temperature only 10 out of 48 districts really did show a significant mortality increase. [67]. Especially if more costly intervention measures are planned, which take proactive approaches to reach the most vulnerable, it is vital to map vulnerable districts in order to be able to allocate prevention measures to those, who really need them. Thus, existing projects for this pursue vulnerability mapping in Germany and elsewhere [68,69].

### 4.6. GPs’ Perceptions of Current and Future Relevance of Heat Health Impacts for the Elderly

So far, there have been few studies on GPs’ perceptions of the relevance of heat health impacts on the elderly. In a representative survey in Germany practice-based physicians attributed a rather low relevance to extreme weather events in general and heat events in particular. These findings are illustrated by a quote from a GP in a qualitative study by Abrahamson et al.: “I had to laugh when I got this and so did the practice manager and so did the deputy practice manager… If you were coming to me talking about the flu epidemic I’d be much more interested.” [51]. In the study at hand, we experienced similar findings for some of the GPs. However, perceptions of relevance differed greatly among GPs and many of them also acknowledged heat to be a health risk for their elderly patients. GPs stressed that the degree of relevance depended on the vulnerability, namely sensitivity determined by individual health conditions and social support and nursing care as well as the nature of the heat exposure. Thus, diverging perceptions of GPs could be attributed to existing differences in quality of care or local climatic differences, for instance higher temperatures in cities due to the urban heat island effect. However, different perceptions are probably not only due to objectively different circumstances, but also to individually different risk perceptions of GPs. Studies have shown, that risk perceptions linked to climate change (but also risk perceptions in general) are highly individual and linked to personal experiences, affects, socio-cultural norms and values [70]. This means, that it is important for GPs to include objective risk evaluations from scientific studies into their decision-making.

Some GPs perceived population aging to be more important to the future relevance of heat health impacts than climate change. Very few studies investigate health professionals’ perception of climate change in particular. Anaker et al. found that climate change is perceived to be important by nurses, but a topic far off health professional’s daily routine [71]. The nurses pointed out that their daily work was centered around the individual patient and was restricted by time and budget constraints, which were more urgently felt than climate change. Moreover, Prasad et al. investigated senior medical students’ perception on climate change and revealed that the urgency to act on climate change was lower in a group primed with a medical case study, suggesting that being in a “medical mode” lowered medical student’s sense of urgency for climate change [72]. These findings by Anaker and Prasad show that the topic of climate change has a rather low priority in the medical setting.

Furthermore, most GPs expressed uncertainty, when talking about climate change (see Figure 3). Uncertainty was found to be a common barrier to engage with the topic of climate change [73,74]. This seems to be true for GPs as well. It is interesting to see, that some GPs attributed an increasing future relevance to heat because of aging populations (sensitivity) only and explicitly not because of climate change (exposure). That might be the case because population aging was not associated with uncertainty and it was a topic GPs are commonly confronted with in their daily routine. Because this argument seems to be one that GPs are ready to follow, it might be worth to emphasize the sensitivity perspective, when promoting heat health prevention measures. On top of that, GPs should be informed about the impacts of climate change as well, so that they understand why adaptation measures like heat health actions plans are necessary. This is supported by Anaker et al., who conclude that increased knowledge of health professionals about climate and environmental issues are the basis to involve health professionals in climate change adaptation and mitigation [71].

While the heat alert system of the German Weather Service had been put into place two years after the heatwave summer of 2003, concrete measures how to convert heat alerts into protective action are still being under development in Germany [75]. For instance, there are no formalized ways to inform GPs about heat warnings. GPs are not obliged to sign up to the heat newsletter offered by the German Weather Service, while nursing homes in Baden-Württemberg are. An evaluation study by Capellaro and Sturm showed that GPs did not receive heat warnings to a considerable extent [76]. To inform the general population, ministries use their websites, press releases and offer information brochures, mainly on their websites. It remains unclear, how effective these passive ways of communication are, especially to reach the elderly population. No active ways of communication as recommended by Lowe et al. [53] are put into place. Although the German Adaptation Plan recommends educating health professionals as multipliers for heat health information since 2011 [77], so far GPs in Baden-Württemberg have not been involved in structured training.

In Baden-Württemberg nursing homes and ambulatory nursing care services are obliged to sign-up for the DWD heat alert newsletter. Furthermore the development of practical guidelines for care-homes during heat waves are planned in the adaptation plan for Baden-Württemberg [78]. However, to our knowledge so far these measures are neither fully implemented nor monitored. In an evaluation study conducted by the Federal Ministry of Environment, Capellaro and Sturm did not have sufficient information to assess whether care homes or hospitals receiving heat warnings adopt any prevention measures. In contrast to that, the federal states of Hesse has implemented action plans for nursing homes and care services as well as regular control mechanisms in heat waves for several years now [79]. Additionally, informal carers and social networks need to be involved for those elderly who do not receive professional care.

## 5. Conclusions

This is one of the first studies investigating GP’s perceptions of heat health impacts on elderly. The presented results lead us to the following recommendations for future research and policy:

As to research, GPs perceptions of heat-related morbidity and mortality diverging from common findings in literature suggest, that more research on health care utilization in heat waves and heat health impacts in the ambulant health care setting is needed. This can help to better understand what kind of support is necessary in the ambulant health care sector. The little acquaintance of GPs with literature in the field of heat health, challenges researchers to foster effective dissemination of their findings in the area of climate change and health to practitioners in the medical field.

As to policy, we draw two main conclusions. First, there is a need to further raising GP’s awareness for the relevance of heat health impacts in order to engage them into preventive action, e.g., within heat health action plans. While announced in the German adaptation strategy long ago, GPs still have not received any training on climate change and health issues including heat health. In order to be able to protect their patients properly and act as mutliplicators, trainings should be integrated in formats commonly attended by GPs, e.g., local quality circles or formational training by the local medical associations (“*Ärztekammer*”). Trainings should frankly discuss the harvesting effect and spatial differences in mortality rates in order to properly address GPs doubts on heat-related mortality. The short duration of health relevant heat events as defined by heat warning systems should be emphasized. In addition, GPs should receive information about climate change projections for Baden-Württemberg, which openly treats uncertainty, but also underlines the strong scientific evidence for increase in temperature and extreme weather events in order to convince GPs that vulnerability is also increasing from the exposure perspective.

Second, GPs emphasize that social support and nursing care for the elderly is one major determinants of vulnerability to heat. Thus, current legislative efforts to strengthen nursing care in Germany (*“Pflegestärkungsgesetze*”) should be pursued further. Additionally, specific measures for nursing care in heat waves, as established in the federal state of Hesse should become standard procedure in Germany in order to achieve a climate resilient health system.

## Figures and Tables

**Table 1:** Drugs associated with increased risks for heat health impacts (adapted from Westaway et al. [15]).

Drug Groups	Possible Effects in Heat Waves
Antidepressants, anticonvulsants, antipsychotics, anticholinergic drugs, diuretics, antihypertensive drugs such as Angiotensin Converting Enzyme (ACE) inhibitors or Angiotensin II Receptor Blockers (ARBs), benzodiazepines, opioids	Impairment of physiological or behavioral adaptation to heat: - Physiological adaptation: e.g., through alterations in sweating, central thermoregulation, thirst sensation, hydration, electrolytes, blood pressure, renal function - Behavioral adaptation: e.g., through sedation, impaired alertness and judgement
Oral antidiabetics, opioids, novel oral anticoagulants, digoxin, lithium	Drug toxicity in dehydrated patients due to reduced renal function

**Table 2:** GPs’ perceptions of current relevance of heat health impacts on the elderly.

GP’s Rationales for Attributed Relevance	Attributed Current Relevance		
	Low Relevance [6/24]	Balanced Attitude [9/24]	High Relevance [9/24]
**Sensitivity:** Individual risk factors Social support and nursing care	- Underlying morbidities outweigh heat impact - Current behavioral adaptation sufficient	- Depends on underlying morbidities and behavioral adaptation of each individual	- Underlying morbidities are aggravated by heat - Insufficient behavioral adaptation
	- Social support and nursing care situation sufficient to prevent heat health impacts	- Depends on care situation which is individually different	- Specific care needs during heat waves, which often can’t be met with current support and nursing care
**Exposure**	- Heat waves in Germany rare and short	- Depends on nature of heat wave, e.g., only relevant for long heat waves	- Local heat exposure especially threatening (e.g., high humidity)

## Supplementary Materials

The following are available online at http://www.mdpi.com/1660-4601/15/5/843/s1.

## Appendix A

**Table A1:** Interview Guide.

Introduction
Greeting and introduction of the interviewer
Repetition of: Topic: Health impacts during heat-waves—possibilities of GPs to protect elderly during heat-waves Goal of the interviews: to investigate GPs’ perception, experiences, ideas on this issue
Definition of terms: Talking about elderly roughly implies geriatric patients, so people above the age of 75, especially with pre-existing diseases and in the need of nursing care.
Repetition of ethical aspects as described and signed beforehand in the informed consent: Live recording on audio tape for transcription Data protection: e.g., original material will not be given to third parties, treated with pseudonyms etc. Personal rights: e.g., chance to stop the interview or withdraw from the study at any time etc.
Note on procedure of interviews: Predominantly open questions, if question was not understood, cordial invitation to clarify
**A. Perceptions of Heat Health Impacts**
**1.** **The topic of my thesis is: Health impacts of heat waves—Possibilities of GPs to protect the elderly. What was you first thought, when you heard of this research project?** **2.** **What is a heat-wave for you?** Explanation given after GP’s answer *: When we talk of heat-waves or heat-periods we speak of at least two consecutive days with a sensed temperature of 32 °C (Trigger of heat-warning by the German Weather Service) **3.** **Which health impacts do you associate with heat waves?** Which health impacts on morbidity and mortality do you associate with heat waves? (Added to interview guide in the course of data acquisition **) **4.** **Imagine taking a general view on the social and health situation of your elderly patients. What relevance do you attribute to heat waves as a risk factor in this general view?** **5.** **Which risk factors promote the possibility that an elderly patient might die during a heat-wave?** Health factors? Social factors? **6.** **Do you think the relevance of this topic will change in the future?** Climate Change?
**B. Perceptions of Heat Health Impact Prevention**
This second part of the interview guide is not displayed here, because results on prevention are not treated in the presented article.

Note: The questions in bold print in Section A were definitely asked, while questions in normal print were only asked, if GPs would not talk about these aspects themselves (prompting). * The explanation in question two was given after GPs had described their own perception of a heat wave in order to have a comparable understanding of heat-waves in the further course of the interview. ** When asked about heat health impacts, GPs seldom associated heat waves with mortality spontaneously. As we noticed this throughout the data collection phase, we specified our question, asking for impacts on morbidity and mortality explicitly in the subsequent interviews.

**Table A2:** GPs’ perceptions of risk factors for their elderly patients to suffer from heat health impacts.

**Pre-existing Disease [23/24]**	**Cardiovascular diseases (CVD) [15]:** CVD in general [6], congestive heart failure [4], high blood pressure [4], arrhythmia [2], coronary heart disease [2], heart attack in patient history [1], stroke [1]
	**Multi-morbidity [8]**
	**Dementia or other cognitive impairment [8]**
	**Respiratory diseases (RD) [6]:** Chronic Obstructive Pulmonary Disease (COPD) [4], RD in general [2], asthma [1]
	**Renal Disease [6]**
	**Diabetes [6]**
	**Psychiatric disorder, e.g., depression [2]**
	**Other impairments:** Electrolyte imbalance [2], acute infections (esp. diarrheal disease) [3], chronic disease in general [2], adiposity [1], collapse in patient history [1], dehydration in patient history [1]
**Socioeconomic Factors [19/24]**	**Social support and nursing care situation [14]:** living alone without support [11], living alone in general [3], living alone or in care homes with insufficient support [2]
	**Housing [7]:** warm housing situation [4], impaired access to logistics [3]
	**Social status [4]:** low financial capacity [3], low education [1]
**Individual Factors [18/24]**	**Age [16]:** Old age [12], young age [4], old age as insufficient criteria [2]
	**Male Sex [1]** (weaker social network)
	**Genetics [3]**
**Intake of Medication [14/24]**	**Cardiovascular medication [13]**: antihypertensive medication in general [8], diuretics [9], beta-blocker [2], calcium antagonists [1], antiarrhythmic agents [1],
	**Other medication [4]:** antibiotics [1], antidiabetics [1], St. John’s wort (photosensitivity) [1], non-prescribed medication (e.g., pain killers) [1].
**Functional Impairments [5/24]**	**Impaired mobility or confinement to bed [5]**
**Alcohol Intake [2/24]**	**Alcohol intake [2]**

**Table A3:** GPs’ perceptions of heat related morbidity (Heat-related diseases mentioned by GPs, coded according to ICD-10).

Groups	Specific Diseases (ICD-10 Coding) [Number of GPs Mentioning It]
**ICD-10 E86 Volume depletion/ E87 Other disorders of fluid, electrolyte and acid-base balance [22/24]**	E86 Volume depletion OR T67.3 Heat exhaustion due to water depletion [18]
	E87 Other disorders of fluid, electrolyte and acid-base balance OR T67.4 Heat exhaustion due to salt depletion [4]
**Circulatory diseases, ICD10-I00-I99 [15/24]**	I99 Other and unspecified disorders of circulatory system [9]: usually naming circulatory collapse and circulatory dysregulation
	I95 Hypotension [4]
	Other [3]: I74 Arterial embolism and thrombosis [1], I64 Stroke, not specified as hemorrhage or infarction [1], I49.9 Cardiac arrhythmia, unspecified [1]
**T67 Effects of heat and light [8/24]**	T67.6 Heat fatigue, transient [5]; T67.0 Heatstroke and sunstroke [3]; T67.5 Heat exhaustion, unspecified [2], T67.1 Heat syncope [2]; T76.7 Heat edema [1]
**Infectious diseases, ICD10-A00-B99 [7/24]**	A00–A09 Intestinal infectious diseases [6]
	Other [3]: B37.2 Candidiasis of skin and nail [1], L00–L08 Infections of the skin and subcutaneous tissue [2], N39.0 Urinary tract infection, site not specified [1]
**Organic mental disorders, ICD-F00-F09 [5/24]**	Disorientation, cognitive or emotional impairment [4] (in line with F05–F07 *)
	Aggravation of dementia [2] (F00–F003)
**Others [5/24]**	N17 Acute renal failure [2]; J98.9 Respiratory disorder, unspecified [1]; L55 Sunburn [1]; Injuries from fall [3]

* F05 Delirium, not induced by alcohol and other psychoactive substances, F06 Other mental disorders due to brain damage and dysfunction due to physical disease, F07 Personality and behavioural disorders due to brain disease, damage and dysfunction.
