# Supplementary figures and images for: General Practitioners’ Perceptions of Heat Health Impacts on the Elderly in the Face of Climate Change—A Qualitative Study in Baden-Württemberg, Germany
Source: Int J Environ Res Public Health. 2018 Apr 24;15(5):843. doi: 10.3390/ijerph15050843 (PMC5981882; doi:10.3390/ijerph15050843)

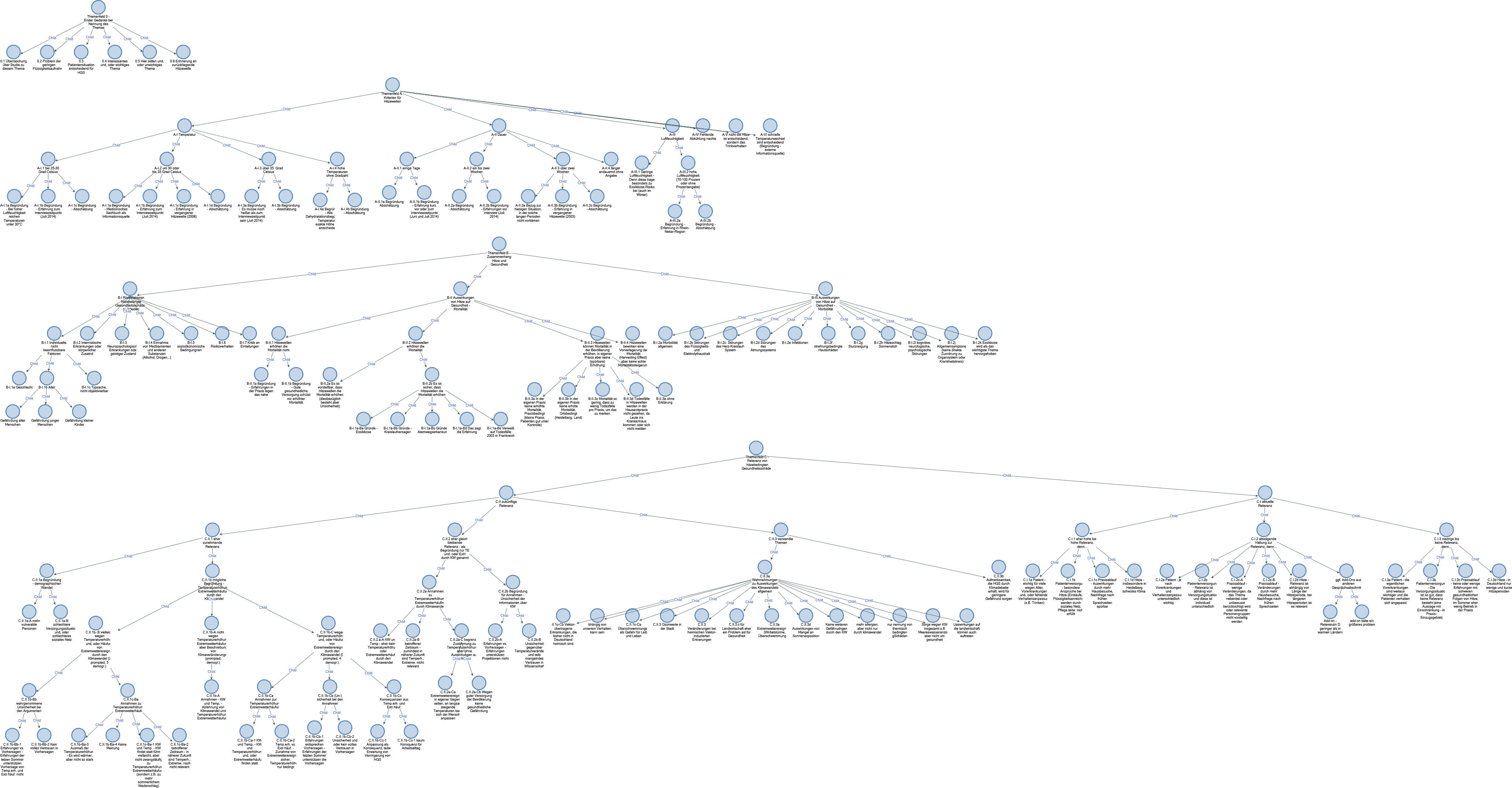

Supplement: Supplementary file 1 [file ijerph-15-00843-s001.zip › GPs' perceptions of HHI_All Codes on all levels.jpg]

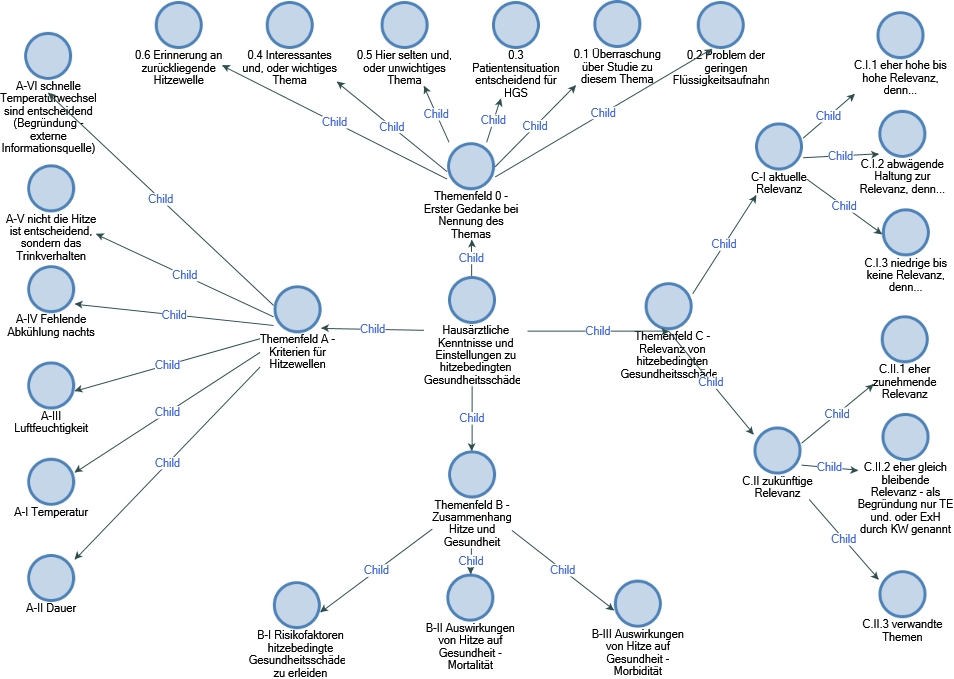

Supplement: Supplementary file 1 [file ijerph-15-00843-s001.zip › GPs' perceptions of HHI_Four themes.jpg]
